# A comprehensive review of sensors of radiation‐induced damage, radiation‐induced proximal events, and cell death

**DOI:** 10.1111/imr.13409

**Published:** 2024-10-19

**Authors:** Saurabh Saini, Prajwal Gurung

**Affiliations:** ^1^ Inflammation Program University of Iowa Iowa City Iowa USA; ^2^ Department of Internal Medicine University of Iowa Iowa City Iowa USA; ^3^ Iowa City Veterans Affairs (VA) Medical Center Iowa City Iowa USA; ^4^ Interdisciplinary Graduate Program in Human Toxicology University of Iowa Iowa City Iowa USA; ^5^ Immunology Graduate Program University of Iowa Iowa City Iowa USA; ^6^ Center for Immunology and Immune Based Disease University of Iowa Iowa City Iowa USA

**Keywords:** cell death, innate immunity, innate sensors, radiation

## Abstract

Radiation, a universal component of Earth's environment, is categorized into non‐ionizing and ionizing forms. While non‐ionizing radiation is relatively harmless, ionizing radiation possesses sufficient energy to ionize atoms and disrupt DNA, leading to cell damage, mutation, cancer, and cell death. The extensive use of radionuclides and ionizing radiation in nuclear technology and medical applications has sparked global concern for their capacity to cause acute and chronic illnesses. Ionizing radiation induces DNA damage either directly through strand breaks and base change or indirectly by generating reactive oxygen species (ROS) and reactive nitrogen species (RNS) via radiolysis of water. This damage triggers a complex cellular response involving recognition of DNA damage, cell cycle arrest, DNA repair mechanisms, release of pro‐inflammatory cytokines, and cell death. This review focuses on the mechanisms of radiation‐induced cellular damage, recognition of DNA damage and subsequent activation of repair processes, and the critical role of the innate immune response in resolution of the injury. Emphasis is placed on pattern recognition receptors (PRRs) and related receptors that detect damage‐associated molecular patterns (DAMPs) and initiate downstream signaling pathways. Radiation‐induced cell death pathways are discussed in detail. Understanding these processes is crucial for developing strategies to mitigate the harmful effects of radiation and improve therapeutic outcomes.

## 1 INTRODUCTION

Radiation is all around us. Naturally occurring radioactive materials are present in the earth's crust, the floors and walls of buildings, food, and even in the human body.^1^, ^2^ Radiation can be broadly divided into two categories: ionizing and non‐ionizing. Non‐ionizing radiation such as radio waves and microwaves have low energy and are harmless. On the other hand, ionizing radiation carries enough energy to remove tightly bound electrons from atoms in living cells, thereby creating ions that damage genetic material (i.e., deoxyribonucleic acids [DNA]). Liberation of tightly bound electrons generates negatively charged free electrons and positively charged ionized atoms. Ionizing radiation includes x‐rays and gamma (γ)‐rays, as well as particles such as alpha and beta particles and neutrons.^3^, ^4^ These rays and particles interact with biological tissues as a function of their varied energy, mass, and charge. X‐rays and γ‐rays have low linear energy transfer (LET) and are highly penetrating electromagnetic waves.^5^ Alpha particles, with high mass and charge, have high LET and cause dense, localized damage but limited penetration, posing risks only if inhaled or ingested.^2^ Beta particles consisting of high‐speed electrons and positrons have moderate LET and penetrate skin but can be stopped by materials such as aluminum. Neutron radiation, which is penetrating but has no charge, causes significant biological damage through nuclear reactions and recoil nuclei formation. Concrete, graphite, heavy water and materials rich in hydrogen are often used to slow down and stop neutrons.

Ionizing radiation and radioactive materials have significant utility in nuclear technology and medicine. Due to developments in research and technology, there has been increased usage of radiation in both fields. In medicine, radionuclides are not only used in radionuclide therapy but also in diagnostics such as x‐ray radiography, positron emission tomography (PET), and single‐photon emission computed tomography (SPECT) to diagnose and treat cancer.^2^ Some standard radionuclides include ^192^Ir, ^125^I, or ^103^Pd used in radionuclide therapy; ^11^C, ^18^F, ^15^O, and ^13^N used in PET; and ^67^Ga, ^111^In, ^123^I, and ^201^Tl used in SPECT.^6^ Indeed, radiopharmaceutical therapy (RPT) has emerged as one of the most effective approaches to treating cancers. This is achieved by linking radioactive isotopes to molecules that specifically target cancer cells.^7^ The radionuclides used in RPT include β‐particle (samarium‐153, lutetium‐177, yttrium‐90, and I‐131) and α‐particle emitters (radium‐223 dichloride).^7^

Although continuous innovation has improved technologies for better diagnostic imaging as well as improved radiation therapy, the side effects of exposure to radiation and radioactive materials remain a major global concern in the medical field.^2^, ^6^ Furthermore, the use of radioactive material by industries to produce nuclear energy and nuclear weapons, as well as the disposal of radioactive waste, poses a high risk to human life.^8^ Nuclear power plants use uranium as the fuel source in nuclear reactors to generate about 20% of electricity for the United States. Nuclear weapons tests conducted during the twentieth century resulted in significant radioactive pollution and the release of radioactive isotopes including ^4^C, ^137^Cs, and ^90^Sr, affecting the atmosphere, aquatic systems, and underground environments. The U.S. and the USSR/Russia were responsible for 82% of all nuclear tests conducted in the atmosphere during 1945–1963 and for 86% of those performed underground during 1951–1992.^9^ Large‐scale nuclear testing contaminated various sites globally (U.S., USSR, U.K., China) and thereby impact the lives of people living nearby (increased rates of thyroid cancer).^9^

Historical incidents and accidents, such as the atomic bombs used during World War II, and nuclear power plant disasters such as Chernobyl (Ukraine), Three Mile Island (U.S.), and Fukushima Daiichi (Japan), have caused mass illness including acute radiation sickness, and death. In addition, long‐term risks of radiation exposure include birth defects, increased risks of cancer and metabolic diseases, psychological effects, and environmental contamination.^10^, ^11^, ^12^, ^13^, ^14^, ^15^ Importantly, both direct and indirect exposure to ionizing radiation can result in acute and chronic illness including cancer, and death.^16^, ^17^ The adverse impact of radiation on the human body has drawn much attention in lay and scientific communities. In this review, we provide an overview of the current state of knowledge regarding the impact of ionizing radiation on cellular responses, as well as the innate sensors that are involved and ensuing cell death.

## 2 IONIZING RADIATION AND DNA DAMAGE

**FIGURE 1:**
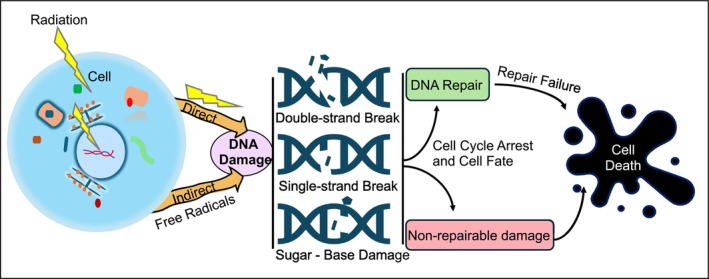
Ionizing radiation‐induced DNA damage and cell fate. Ionizing radiation can promote DNA damage directly by radiation‐induced DNA break, or indirectly via the generation of free radicals. Ionizing radiation promotes single‐strand break, double‐strand break, and base and sugar damage in DNA strands. This DNA damage triggers the cell to arrest its cycle, leading to a decision point: The cell may either initiate repair pathways or undergo cell death.

Ionizing radiation damages cellular DNA, either directly or indirectly (Figure 1). Radiation exposure directly causes single‐ and double‐strand DNA break, cluster DNA damage, and DNA base damage. Indirectly, ionizing radiation causes radiolysis of water and generates free radicals, which have detrimental effects on the exposed cells.^18^ These free radicals can be converted into reactive oxygen species (ROS), hydroxyl radicals (^•^OH), peroxyl radicals (ROO‐), hydrogen peroxide (H_2_O_2_), and reactive nitrogen species (RNS).^19^, ^20^ Thus, radiation produces an array of lesions that include single and double strand break and base alteration in DNA through oxidative damage.^21^, ^22^ In addition to the nucleus, mitochondrial DNA is likely to be a critical target of ionizing radiation. The reactive species, ROS and RNS, alter mitochondrial function, damage mitochondrial DNA, and induce cell death by targeting cell survival‐related genes.^23^, ^24^

The responses of vascular, parenchymal, and connective tissues to radiation exposure varies depending on organ and cell type. Acute responses such as swelling, changes in vascular permeability, edema, and cell infiltration occur rapidly, often within an hour.^25^ Chronic effects occur months to years after exposure, culminating in fibrosis, organ failure, and necrosis.^25^ Radiation‐induced cell lesions are recognized by specific sensors that activate cellular responses to restore homeostasis. However, if the cell's repair system collapses and fails to repair DNA damage, cells die by different modalities of cell death (described below) or enter senescence/cell cycle arrest, ultimately undergoing secondary apoptosis.^21^, ^26^

Recognition of radiation‐induced cellular damage involves both innate and adaptive immunity. The innate immune response provides the initial defense against invading pathogens or tissue injury by recognizing pathogen‐associated molecular patterns (PAMPs) and damage‐associated molecular patterns (DAMPs) via pattern‐recognition receptors (PRRs).^27^ In the context of radiation‐induced damage, PRRs that specifically recognize damaged DNA trigger downstream biologic responses via activation of proximal signal transduction pathways.^28^ Biologic responses such as the production of cytokines and chemokines by immune cells following radiation play a critical role in modulating the immune response to radiation.^29^, ^30^

## 3 CELLULAR RESPONSES TO RADIATION

The cellular response to radiation is complex and dynamic, involving various cellular and molecular mechanisms. Ionizing radiation is one of the most substantial external agents that induce DNA double strand breaks, which represents the most critical biological damage induced by radiation.^31^, ^32^ Cells that divide slowly or are quiescent, such as those found in the nervous system, exhibit lower radiosensitivity. Cells that proliferate rapidly, such as those in bone marrow, gastrointestinal tract, and skin, are more radiosensitive.^5^ When cells are exposed to ionizing radiation, such as x‐rays or γ‐rays, they undergo a series of molecular and cellular events to sense and repair DNA damage, thereby maintaining cell viability.^33^, ^34^, ^35^ Herein, we discuss the key aspects of cellular responses to radiation exposure.

### 3.1 DNA damage recognition

**FIGURE 2:**
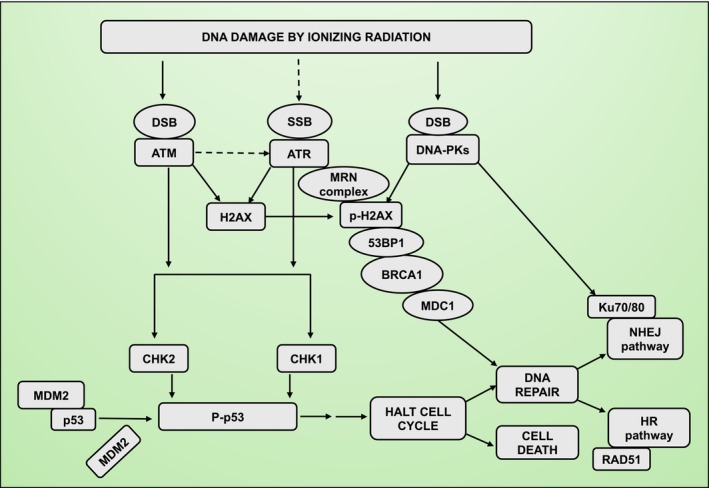
Pathways of DNA damage recognition and cell fate determination following ionizing radiation. Ionizing radiation causes DNA damage, including single‐strand break (SSB) and double‐strand break (DSB). The primary DNA damage sensors involved are the PIKK family kinases: ATM, ATR, and DNA‐PKcs. These kinases are activated in response to DSBs and SSBs, initiating the DNA damage response (DDR). Phosphorylation of H2AX by PIKK kinases (ATM, ATR, and DNA‐PK) at serine 139 forms γ‐H2AX foci, marking the damage site for recruitment of repair proteins. Upon activation, ATM and ATR phosphorylate CHK1 and CHK2, leading to the stabilization and activation of p53 by displaced MDM2. The interaction between ATM, ATR, DNA‐PKcs, and downstream effectors such as CHK1/CHK2 and p53 determines whether the cell will halt the repair cycle or initiate apoptosis if the damage is beyond repair. The pathways for repair include non‐homologous end joining (NHEJ) and homologous recombination (HR).

Cells utilize intricate mechanisms to recognize and repair DNA damage (Figure 2).^36^ This involves members of the phosphatidyl inositol 3′ kinase‐related kinase (PIKK) family including ataxia‐telangiectasia mutated (ATM), ataxia‐telangiectasia and Rad3‐related (ATR), and DNA‐dependent protein kinases (DNA‐PK), well‐known protein complexes recruited to sites of DNA damage.^37^ These kinases are also known as the “trinity” at the core of DNA damage response due to their central roles in detection of DNA damage, activation of repair pathways, and maintenance of genomic stability.^37^ Activation of ATM occurs within 10 min of radiation exposure, followed by ATR foci formation between 60 and 120 min.^38^ Adams et al. showed that the expression and activation kinetics of ATM and ATR are consistent across different cell types, including HeLa and Swiss 3 T3 cells, and are not dependent on radiation dose (0.5 Gy vs.10 Gy).^38^ Furthermore, the MRN complex (proteins of the checkpoint pathway; Mre11, Rad50, and Nbs1) is required for ATR formation. The recruitment of ATR and its downstream signaling to activate CHK1 at a DNA damage site require the kinase activity of ATM.^38^ However, decreased expression of ATR or CHK1 does not affect ATM activation.^38^, ^39^

Interactions between ATM, ATR, and DNA‐PKs in regulation of the DNA damage checkpoint during the G2 phase of the cell cycle are radiation dose‐dependent.^39^ Mladenov et al. showed that at a 2 Gy dose, ATM and ATR kinases are both essential and function together in the DNA damage checkpoint during the G2 phase of the cell cycle in 82‐6hTert and human lung carcinoma A549 cell lines.^39^ The molecules function in a linear module, where ATM is upstream and ATR downstream, primarily interfacing with the cell cycle machinery through CHK1.^39^ But at a 10 Gy radiation dose, cooperation between ATM and ATR becomes uncoupled, and both kinases function independently to maintain a checkpoint response that leads to cell cycle arrest.^39^ DNA‐PKcs‐deficient cells (M059J, HCT116) show hyperactivation of G2 phase checkpoint following low and high radiation doses (1–10 Gy), demonstrating a role for DNA‐PKcs in ATM/ATR‐mediated G2 phase checkpoint.^39^ This reflects crosstalk between DNA‐PK and ATM/ATR kinase during radiation‐induced DNA damage.^39^

Histone H2A family molecules play a central role in double‐stranded DNA damage repair. The histone variant H2AX phosphorylation is a key marker of DNA double strand break, and facilitates the recruitment of repair machinery to damaged sites playing a crucial role in repair.^18^ After radiation exposure, H2AX undergoes rapid phosphorylation in response to double strand break, specifically at the serine 139 site, forming γ‐H2AX foci, which is a common substrate of the PI3K family of proteins.^40^, ^41^ This modification is a crucial early step in the DNA damage repair response, facilitating the recruitment of repair proteins to the damage site. The γ‐H2Ax forms in distinct domains around the DNA break depending on chromatin contact and organization.^40^ It enhances the effectiveness of DNA repair and decreases sensitivity to radiation. It helps keep double‐strand break ends in proximity, preventing large chromosomal losses. These domains are critical for the organization of the DNA repair process.^41^ The peak level of γ‐H2AX is observed within 15–30 min following radiation exposure, and the extent of H2AX phosphorylation (number of γ‐H2AX foci) correlates with cellular radiosensitivity.^42^ Normal tissue cells, human SiHa cervical carcinoma cells, and more radiosensitive SCCVII tumor cells tend to have a higher number of γ‐H2AX foci shortly after 10–20 Gy irradiation and show a slower decline in γ‐H2AX foci over time, indicating impairment of DNA repair mechanisms.^42^

PIKK kinases mediate H2AX phosphorylation in response to DNA damage caused by ionizing radiation.^43^ ATM and DNA‐PK kinases have redundant functions, and thus, both can phosphorylate H2AX on serine 139 to create γ‐H2AX.^44^ H2AX phosphorylation occurs effectively in the primary fibroblast cell lines lacking ATM (AT1BR and AT7BI) and in the DNA‐PK‐deficient glioma (M059J) human cell line^44^ This has also been observed in irradiated mouse embryonic fibroblast (MEFs) cells deficient in ATM or DNK‐PKs.^44^ In another study, H2AX phosphorylation was impaired in HeLa cells exposed to 10 Gy radiation in the presence of wortmannin (PI3K and PIKK inhibitor; i.e., inhibits both ATM and DNA‐PK at higher concentration used in the study).^45^ In support of redundant function of these kinases, radiation‐induced H2AX phosphorylation was reduced in ATM‐deficient MEF cells when treated with LY294002 (a PI3K specific inhibitor that can also inhibit DNA‐PK activity).^44^ Wang et al. observed that initial H2AX phosphorylation is delayed when ATM alone is inhibited by caffeine in DNA‐PK deficient cells.^45^ Importantly, phosphorylation of H2AX is still detected under all conditions of genetic or chemical inhibition, suggesting the redundant function of additional kinases such as ATR. Taken together, these studies highlight the redundancy in PIKK kinase family proteins in H2AX phosphorylation following radiation‐induced DNA damage.

### 3.2 Cell cycle arrest

In response to radiation‐induced DNA damage, cells activate cell cycle checkpoints to halt cell cycle progression and allow time for DNA repair. Fluorescence time‐lapse microscopy has been used to study the dynamics of DNA damage checkpoints in G1, S, and G2 phases by observing cell‐cycle progression after acute DNA damage in single cells.^46^ The G1 checkpoint completely halts progression but is more permissive to DNA damage. The S phase checkpoint is the least sensitive, slowing but not stopping progression. The G2 checkpoint is the strictest, completely halting progression in response to damage. The timing of DNA damage within each phase influences the sensitivity and outcome of the checkpoint responses.

ATM and ATR act as “early responders” to double strand breaks and replication stress from single‐strand damage and intra‐ and inter‐strand crosslinks (Figure 2). Upon activation of PIKKs, extensive networks of proteins, including the downstream effector kinases CHK1 and CHK2, are activated.^47^ These kinases phosphorylate p53 and other targets to halt the cell cycle in response to DNA damage, facilitate DNA repair, and trigger programmed cell death pathways if the damage is irreparable. Under normal conditions, p53 levels are kept low and inactive due to the p53 complex with MDM2, which promotes ubiquitin‐dependent proteolysis of p53. DNA damage triggers a series of phosphorylation, dephosphorylation, and acetylation events of p53.^48^ The phosphorylation of p53 occurs at serine 15 and serine 20, displacing MDM2 and relieving p53 from proteasomal degradation. Stabilization and prevention of p53 degradation are very important during the repair of radiation‐induced DNA damage. In lymphoblast cells, 10 Gy radiation induced activation of DNA‐PK and AKT/PKB, and inactivation of GSK‐3 and MDM2, all of which are required for activation and stabilization of p53.^49^ Both DNA‐PK and AKT/PKB phosphorylate p53 following radiation, which prevents p53 degradation and allows it to accumulate and function effectively in the nucleus.^49^ P53 activation enhances its DNA‐binding and transcriptional abilities and promotes activation of genes that cause cell‐cycle arrest, apoptosis, or DNA repair.^48^

These cell cycle checkpoints are vital for detection and response to DNA damage caused by radiation, thereby maintaining genome stability. By halting the cell cycle, these checkpoints prevent cells with damaged DNA from entering mitosis.^50^ Thus, cell cycle checkpoints act as safeguards against the development of cancer, and when the DNA damage is too severe to be repaired, these checkpoints can trigger programmed cell death or cellular senescence (a state of permanent growth arrest).^50^

### 3.3 DNA repair

The two major repair pathways, non‐homologous end joining (NHEJ) and homologous recombination (HR), are involved in repairing DNA double strand break (Figure 2). NHEJ is an error‐prone mechanism and is often used when a quick repair is needed for double‐strand break identification and end protection. DNA‐PK promotes NHEJ by direct ligation of the DNA ends to seal the break.^51^ In contrast, HR is a highly efficient and error‐free method of DNA repair for restoring chromatin integrity and functionality. However, HR is only available after DNA replication during the S and G2 phases of the cell cycle when a homologous DNA sequence can be utilized as a template for DNA synthesis to replace broken DNA.^52^ During radiation‐induced damage, RAD51 facilitates the detection of breaks and insertion of the homologous DNA sequence enabling accurate repair by HR.^53^

The availability and activity of repair proteins such as Ku70/80 (involved in NHEJ) and BRCA1/2 and RAD51 (involved in HR) play a crucial role in determining which repair pathway is used. Importantly, if proteins important for one pathway are mutated or deficient, cells may rely on the alternative repair mechanisms.^53^

The efficiency and fidelity of DNA repair mechanisms determine the cell's ability to survive and maintain genomic stability following irradiation. DNA‐PK is a central player in the NHEJ pathway, which repairs double‐strand breaks by directly ligating the broken DNA ends. Mi and colleagues showed that activation of DNA‐PK is crucial for initiating DNA NHEJ, after ionizing radiation induces DNA damage.^54^ They reported a significant increase in the association of endogenous DNA‐PKcs with PP6R1 and PP6c in DNA‐PK proficient (M059K) glioblastoma cells following radiation (5–10 Gy). Upon ionizing radiation‐induced DNA damage, PP6 interacts with DNA‐PKcs, leading to its dephosphorylation and subsequent activation. This enables DNA‐PKcs to bind to DNA ends and form a complex with the Ku70/Ku80, initiating the NHEJ repair process. Similar studies in human glioblastoma cells showed a role for DNA‐PK in double‐strand break repair by NHEJ following high‐ to low‐LET radiation (gamma rays, alpha particles, and high‐charge and energy [HZE] ions).^55^ In the absence of DNA‐PKcs activity, reduced double‐strand break repair and increased recruitment of RAD51 were seen at 24 h post‐radiation.^55^

A recent study by Fang et al. showed that RecQ‐mediated genome instability protein 1 (RMI1) is low in normal cell lines (IMR90 and MRC5) and relatively high in cancer cells (U2OS, HeLa, and H460). Furthermore, radiation upregulates RMI1 protein levels and its relocation to DNA damage sites, where RMI1 along with RAD51 promote HR‐mediated DNA repair.^56^ During exposure to ionizing radiation exposure (4 and 8 Gy), RMI1 prevents and resolves abnormal recombination products during HR to maintain genomic stability in both normal and cancer cell lines.^56^

Thus, cellular responses to acute radiation are tightly regulated and interconnected, aiming to maintain genomic stability, repair DNA damage, eliminate severely damaged cells, and initiate tissue repair processes. The balance between cell survival and cell death pathways determines the outcome of radiation exposure, impacting tissue integrity and the overall radiation response.

## 4 INNATE IMMUNE SENSORS

**FIGURE 3:**
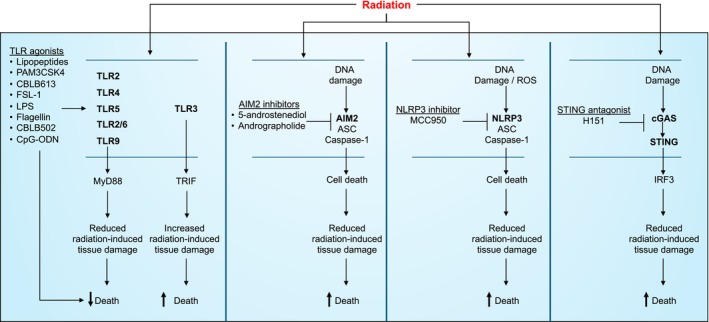
Roles of different pattern recognition receptors in radiation‐induced toxicity. Pattern recognition receptors Toll‐like receptors: TLRs 2, 4, 5, 2/6, and 9 signaling inhibit radiation‐induced tissue damage and play protective roles during radiation‐induced morbidity and mortality. As such, TLR agonists have been shown to have both radioprotective and radiomitigative effects in acute radiation syndrome. The protective effects of these TLRs are mediated via MyD88. In contrast, TLR3‐TRIF signaling is pathogenic during radiation. AIM2: Cytoplasmic sensor AIM2 senses radiation‐induced damaged DNA, which promotes cell death and ultimately contributes to acute radiation syndrome. As such, AIM2 inhibitors have been shown to provide protection from radiation‐induced lethality in mice. NLRP3: Cytoplasmic sensor NLRP3 senses radiation‐induced DNA damage/ROS, which promotes cell death and contributes to radiation‐induced lethality in mice. Inhibition of NLRP3 by MCC950 (a potent NLRP3 inhibitor) protects mice from radiation‐induced toxicity in vivo. cGAS/STING: DNA sensor cGAS senses radiation‐induced DNA damage, which ultimately activates STING. STING activation results in IRF3 activation, which promotes radiation‐induced tissue damage and toxicity. In support of the pathogenic role for STING, STING antagonist H151 protects mice from acute radiation syndrome.

The innate immune system plays a key role in recognizing and triggering inflammation in response to microbial infection or tissue damage. Recognition is mediated by germline‐encoded pattern‐recognition receptors (PRRs) that bind pathogen‐associated molecular patterns (PAMPs) and damage‐associated molecular patterns (DAMPs).^57^, ^58^ PRRs can be categorized into five types based on the similarity of their protein domains: toll‐like receptors (TLRs), C‐type lectin receptors (CLRs), nucleotide‐binding oligomerization domain‐like receptors (NLRs), retinoic acid‐inducible gene I‐like receptors (RLRs), and absent in melanoma 2‐like receptors (ALRs).^59^, ^60^ TLR and CLR are membrane‐associated receptors, and as such recognize extracellular and endosomal PAMPs and DAMPs. NLR, RLR, and ALR are cytoplasmic sensors and thus recognize intracellular PAMPs and DAMPs. When these PRRs are activated, they trigger downstream signaling pathways such as NFκB, MAPK, TBK1‐IRF3, and inflammasome signaling. This activation leads to the production of pro‐inflammatory cytokines such as IL‐1β, IL‐6, and tumor necrosis factor (TNF).^59^, ^61^ DAMPs produced upon radiation exposure are recognized by innate immune sensors (Figure 3).^62^

### 4.1 Toll‐like receptors (TLRs)

TLRs are one of the main type of sensors that recognize PAMPs or DAMPs on the cell surface and in endosomes. They can be broadly classified into two subgroups based on their cellular localization and the specific PAMPs they recognize. The first subgroup includes TLRs 1, 2, 4, 5, 6, and 11, which are located on the cell surface and primarily detect microbial membrane components including lipids, lipoproteins, and proteins. The second subgroup consists of TLRs 3, 7, 8, and 9, which are found exclusively within intracellular vesicles, such as the endoplasmic reticulum (ER), endosomes, lysosomes, and endo‐lysosomes where they recognize microbial nucleic acids.^63^ Recognition of cognate PAMPs and DAMPs results in TLR dimerization and, subsequently, cytoplasmic domain recruitment of either TRIF or MyD88.^64^ Of note, all TLRs except TLR3 utilize MyD88 to activate downstream signaling cascade. TLR3 specifically utilizes TRIF to activate downstream signaling, whereas TLR4 utilizes both MyD88 and TRIF.

Studies examining the effects of radiation on TLRs are incomplete, as the effects of radiation on only a few TLRs have been examined. A study by Yoshino et al. showed that x‐ray irradiation increased TLR2 and TLR4 protein expression on THP‐1 monocytes in a dose‐dependent manner.^65^ Interestingly, they failed to see similar upregulation of TLR2 and TLR4 expression when THP‐1 macrophages were exposed to similar doses of x‐ray radiation; rather, x‐ray radiation reduced TLR2 and TLR4 protein levels in THP‐1 macrophages.^65^ In another study, radiation‐induced upregulation of TLR2 and TLR4 on THP1 monocytes was rescued by N‐acetyl‐L‐cysteine (NAC), a ROS inhibitor.^66^ Thus, radiation‐induced ROS accumulation in THP1 monocytes promotes upregulation of TLR2 and TLR4. Paun et al. reported the role of these TLRs in in vivo 18 Gy whole thorax radiation of WT, TLR2‐deficient, TLR4‐deficient, and TLR2 × TLR4‐double C57BL/6 deficient mice.^67^ TLR2 and TLR4‐single‐deficient mice succumbed to 18 Gy whole thorax radiation with similar kinetics when compared to WT controls.^67^ TLR2 × TLR4‐double deficient mice were highly susceptible to 18 Gy whole thorax radiation and died with significantly earlier kinetics when compared to WT controls. In addition, TLR2 × TLR4‐double‐deficient mice presented with overall enhanced fibrosis of the lungs when compared to WT controls. In a separate study, TLR2‐deficient mice were found to be highly susceptible to 6.5, 7.5, and 8.5 Gy radiation and displayed greater mortality rates compared to WT controls.^68^ Likewise, compared to control mice, TLR4‐deficient mice were found to have reduced survival when subjected to 5, 7, or 9 Gy radiation. These studies demonstrate a protective role for TLR2 and TLR4 in radiation‐induced morbidity and mortality. Given that both TLR2 and TLR4 signal via MyD88, it could be expected that MyD88‐deficiency provides similar protection from radiation. Indeed, MyD88‐deficient mice were highly susceptible to 6.5 Gy whole body and 14 Gy whole thorax radiation and displayed increased morbidity and mortality when compared to WT controls.^68^, ^69^ Examination of irradiated lung tissues ex vivo demonstrated that MyD88‐deficient lungs had significantly increased cellular infiltration, fibrosis, collagen deposition, and chronic lung injury when compared to WT lungs.^69^ These studies highlight a role for the TLR‐MyD88 signaling axis in providing protection from radiation‐induced morbidity and mortality.

In support of protective roles for TLRs during radiation‐induced morbidity and mortality, several studies have shown that TLR agonists can provide radio‐preventive and radio‐mitigative effects, and several TLR agonists are currently being developed as radiation countermeasures for acute radiation syndrome.^70^ TLR5 in particular has been targeted for its protective role. Burdelya et al. showed that pre‐treatment of mice with purified flagellin (0.2 mg/kg) from Salmonella provided almost complete protection from 10 to 13 Gy whole body radiation (WBR).^71^ While 100% of control mice died in 10 and 13 Gy irradiated groups, flagellin pretreatment resulted in 100% survival in 10 Gy groups and ~80% survival in 13 Gy irradiated groups. Similar results were observed by another independent study wherein 50 μg flagellin provided complete protection from 8 Gy radiation in WT C57BL/6 mice.^72^ More importantly, similar results were observed with CBLB502 (entolimod), a TLR5 agonist derived from Salmonella flagellin. Treatment of mice with CBLB502 (0.2 mg/kg) before 13 Gy WBR significantly enhanced survival compared to PBS controls, which all succumbed to radiation‐induced lethality.^71^ More impressively, CBLB502 administered to rhesus macaques at 0.4 mg/kg 45 min before 6.5 Gy WBR enhanced survival compared to PBS‐treated rhesus macaques.^71^ Subsequent studies have shown that a single dose of CBLB502 (10–40 μg/kg) given within 48 h of 6.5 Gy radiation provides superior protection in non‐human primates.^73^

Similarly, TLR2 stimulation has also been shown to provide protection from radiation. Colonization of C57BL/6 WT mice with *Lactobacillus rhamnosus* (pretreatment with 3 days of oral gavage daily) provides significant protection from 12 Gy WBR in a TLR2‐dependent manner.^74^ Synthetic lipopeptides that mimic the structure of bacterial lipoproteins activate TLR2. Pretreatment with these synthetic lipopeptides (3 mg/mouse) enhances survival following 9Gy WBR in mice.^75^ Pretreatment of WT mice with a single dose of Pam_3_CSK_4_ (a TLR2 agonist; 50 ng/mouse) enhanced survival when subjected to 7.5, 8.5, and 9.5 Gy WBR compared to PBS‐treated controls.^68^ Activation of TLR4 with 20 μg LPS prior to 8 Gy WBR resulted in 100% survival of C57BL/6 mice, and all untreated controls died.^72^ Similarly, mice treated with 2.5 mg/kg LPS displayed significant survival advantage during 7, 9, and 13 Gy radiation compared to PBS controls.^76^ More recent studies have used TLR2/6 agonists CBLB613 (a natural lipopeptide of *Mycoplasma arginine*) and fibroblast‐stimulating lipopeptide‐1 (FSL‐1) as potential targets to provide protection from radiation‐induced morbidity and mortality. CBLB613 given at a dose as low as 0.025 mg/kg to mice before or after 9.2 Gy radiation provided significant survival advantage over PBS controls.^77^ Likewise, a single dose of 0.25 mg/kg FSL‐1 given 24 h after 9.2 Gy WBR enhanced survival and mitigated clinical score increases and weight loss when compared to no‐treatment controls, demonstrating a mitigative role for FSL‐1 during acute radiation syndrome.^78^, ^79^

Treatment with 50 μg CPG‐ODN to activate TLR9 in BALB/c mice provided significant protection from lethal WBR.^80^, ^81^ The survival enhancement effects of CPG‐ODN pretreatment over untreated controls were observed in BALB/c mice exposed to 6.5, 8, or 10 Gy radiation.^80^, ^81^ Importantly, TLR9 agonist (1 mg/kg dose) provided significant protection from 8.4, 9.4, and 10.4 Gy radiation in C57BL/6 mice, regardless of whether the agonist was administered 1 h before or 1 h after radiation.^82^

In contrast to other TLR studies, Takemura et al. reported that 10 Gy WBR exposure in TLR3‐deficient mice showed significantly improved morbidity as demonstrated by milder weight loss and diarrhea, as well as delayed death, suggesting a pathogenic role for TLR3 during radiation‐induced toxicity.^83^ While all WT mice succumbed to a radiation‐induced gastrointestinal syndrome model (10 Gy WBR followed by isologous bone marrow transplantation) by day 8, but all TLR3‐deficient mice survived. In the same study, TRIF‐deficient mice survived significantly longer than controls after 10 Gy WBR, highlighting a pathogenic role for the TLR3‐TRIF pathway in radiation‐induced toxicity. Unlike other TLRs, TLR3 only signals through TRIF.

Taken together, these studies highlight a clear protective role for TLR‐MyD88 signaling in acute radiation syndrome. As reviewed above, stimulation of TLRs 2, 4, 2/6, 5 and 9 prior to or after lethal radiation can enhance the survival of mice. Thus, TLR agonists that activate MyD88 signaling may be used as both preventive and mitigative agents for alleviating radiation‐induced toxicity. In contrast, TLR3‐TRIF signaling exacerbates acute radiation syndrome. In future studies, it will be important to examine what signaling aspects downstream of MyD88 and TRIF promote these counteractive outcomes to radiation.

### 4.2 AIM2‐like receptors (ALRs)

Absent in melanoma 2 (AIM2)‐like receptors (ALRs) are pattern recognition receptors characterized by the presence of a DNA‐binding HIN‐200 and Pyrin domain. AIM2 is one of the best‐studied receptors in the ALR family and is known for its ability to recognize cytoplasmic double‐stranded DNA. Upon recognition, AIM2 recruits adapter protein ASC (apoptosis‐associated speck‐like protein containing a CARD) and protease caspase‐1 to form a multimeric protein complex known as AIM2 inflammasome. AIM2 inflammasome activation results in caspase‐1‐mediated production of mature IL‐1β and IL‐18, as well as pyroptosis, a lytic form of cell death.^84^, ^85^ However, AIM2 also has inflammasome‐independent roles, specifically in the setting of colitis and colorectal cancer, where AIM2 attenuates colon cancer development by regulating stem cell proliferation, primarily by inhibiting excessive AKT activation.^86^, ^87^

Bo et al. demonstrated that AIM2 co‐immunoprecipitates with γH2AX in a mouse model.^88^ AIM2 was demonstrated to be in proximity to γH2AX foci in the nucleus using immunofluorescence microscopy. γH2AX foci are sites of DNA double‐strand breaks that occur following radiation exposure of primary bone marrow‐derived macrophages. These data demonstrate a previously unknown role for AIM2 in sensing damaged self‐DNA within the nucleus. Furthermore, AIM2 formed an inflammasome complex in the nucleus, as AIM2 specks containing ASC were detected in the nucleus following radiation of BMDM in vitro. In vivo radiation exposure experiments demonstrated increased survival following 7 Gy WBR in AIM2‐deficient mice, compared to WT controls. Similar protection of AIM2‐deficient mice was observed when WT and AIM2‐deficient mice were subjected to 14.2 Gy subtotal body radiation (gut‐specific radiation). Radiation of mice deficient in IL‐1β, IL‐1R, or IL‐18 failed to provide any survival advantage over WT controls, suggesting a role for pyroptosis. Radiation‐induced AIM2 inflammasome activation results in caspase‐1 activation – which subsequently promotes maturation of IL‐1β, IL‐18, and pyroptotic cell death in BMDM. In support of a pathogenic role for AIM2 in acute radiation syndrome, inhibition of AIM2 inflammasome by 5‐androstenediol (a natural steroid hormone produced in the adrenal cortex) during 8, 9, and 10 Gy WBR enhanced survival and delayed death.^89^ Furthermore, inhibition of AIM2 by andrographolide (an active compound isolated from *Andrographis paniculate*) improved survival of WT mice exposed to 18 Gy whole thorax radiation in a model of radiation‐induced lung injury.^90^ These studies argue for a pathogenic role of AIM2 in acute radiation syndrome. However, a more recent study using AIM2 littermate controls failed to observe a similar pathogenic role for AIM2 during acute radiation syndrome.^91^ Compared to non‐littermate WT controls or AIM2‐WT littermates, AIM2‐heterozygous and AIM2‐knockout mice showed reduced survival following 8 Gy WBR exposure. Thus, the role of AIM2 in acute radiation syndrome in vivo remains unresolved.

### 4.3 NOD‐like receptors (NLRs)

NLRs are one of the largest groups of cytoplasmic PRR, known for detecting a wide range of pathogenic and non‐infectious stimuli within the intracellular niche.^92^ There are 23 NLRs in humans and 34 in mice that have been identified to date. Structurally, all NLRs consist of a common central nucleotide oligomerization domain, an N‐terminal effector domain, and a C‐terminal leucine‐rich repeat.^93^ NLRs are classified into subgroups based on N‐terminal effector domain differences, and include NLRA, NLRB, NLRC, NLRP, and NLRX proteins.^60^ Functionally, NLRs can be broadly subdivided into four groups: NLRs that activate signaling pathways, NLRs that inhibit signaling pathways, NLRs that act as transcription factors, and NLRs that form inflammasomes.^93^ Multi‐protein inflammasome complexes activate the proteolytic cleavage of caspase‐1. Active caspase‐1 promotes cleavage and maturation of IL‐1β and IL‐18 into their bioactive forms and cleavage of the pore‐forming protein gasdermin D (GSDMD), resulting in pyroptosis.^60^ The role of NLR in radiation remains understudied, and so far, studies have only examined the role of NLRP3 in detail. In this section, we will discuss the current state of our understanding on the role of NLRP3 during in the response to radiation.

NLRP3‐deficient mice are significantly more resistant to a lethal 9.5 Gy WBR than WT, displaying delayed death and overall greater survival.^94^ Mechanistically, radiation promotes NLRP3 inflammasome activation as demonstrated by caspase‐1 activation and production of pro‐inflammatory cytokines IL‐1β and IL‐18 both in vitro in BMDM and in vivo in the spleen of irradiated mice.^94^ Similarly, radiation promotes NLRP3 inflammasome activation in primary Kupffer cells.^95^ In a model of radiation‐induced liver damage, NLRP3‐deficient mice were protected from liver damage as demonstrated by attenuated liver steatosis and reduced levels of serum ALT and AST.^95^ Treatment with MCC950 (a potent NLRP3 inhibitor, 50 mg/kg dose) was shown to ameliorate radiation‐induced mortality in mice.^96^ Importantly, MCC950 was administered 24 h after 9.5 WBR, revealing a radio‐mitigative effect of NLRP3 inhibitors. In a model of radiation‐induced lung damage, NLRP3‐deficient mice showed reduced cellular infiltration of the lungs and production of pro‐inflammatory cytokines in the bronchoalveolar lavage, including IL‐1β and IL‐18.^97^, ^98^ Likewise, treatment with MCC950 at doses of 5 and 10 mg/kg attenuated radiation‐induced lung inflammation and cytokine production.^97^ These studies highlight a pathogenic role for NLRP3 in promoting radiation‐induced tissue damage and lethality.

Interestingly, another study using a conditional deletion of NLRP3 in the myeloid compartment (NLRP3‐flox x Lyz2‐Cre mice) observed a completely opposite role for NLRP3.^99^ Mice lacking NLRP3 in the myeloid compartment died with earlier kinetics and demonstrated overall increased death following 14 or 16 Gy subtotal abdominal radiation. Moreover, mice lacking NLRP3 in the myeloid compartment also displayed greater levels of radiation‐induced skin ulcers than WT controls. The increased death rate in NLRP3‐deficient mice was associated with overall increased colon pathology as assessed macroscopically and by H&E staining. Thus, in the myeloid compartment, NLRP3 may play a protective role during radiation‐induced tissue damage and lethality.

In a contrasting finding, NLRP3‐deficient and WT mice showed no significant difference in caspase‐1 activation or cell death following WBR exposure, demonstrating that NLRP3 is likely not involved in the response to radiation‐induced damage.^100^ Moreover, two independent studies have demonstrated that neither NLRP3 nor NLRC4 are required for radiation‐induced lethality, as NLRP3‐ and NLRC4‐deficient mice did not succumb to lethal WBR compared to WT and littermate controls.^88^, ^91^

These studies highlight that radiation exposure activates the NLRP3 inflammasome in various cell types in vitro and in vivo. Resolution of the protective versus pathogenic roles of NLRP3 during the response to radiation may depend on studies using cell type‐specific NLRP3 deficiency (i.e., whole‐body KO vs. conditional KO mice) and, potentially, microbiota. Future studies that carefully evaluate all these nuanced aspects will hopefully resolve current discrepant results regarding the role of NLRP3 in the response to radiation.

### 4.4 Cyclic GMP‐AMP synthase (cGAS) and STING

cGAS is a cytosolic DNA sensor that detects double‐stranded DNA and responds by production of cyclic GMP‐AMP (cGAMP), which in turn activates stimulator of interferon genes (STING).^101^, ^102^ The role of cGAS/STING in sensing of tumor DNA during radiotherapy has been studied^103^; however, the role of these DNA sensors in radiation‐induced tissue damage and acute radiation syndrome is only emerging. Herein, we will discuss recent advancements in our understanding the role of these sensors in the response to radiation exposure.

In a mouse model of radiation‐induced lung injury, cGAS and STING levels were significantly upregulated in the lungs post‐radiation.^98^, ^104^ More importantly, phosphorylation of STING, TBK1, and IRF3 was all increased in the lungs on day 7 post‐radiation compared to WT control lungs.^98^, ^104^ Both cGAS and STING‐deficient mice showed delayed death and greater survival following 16 Gy whole thoracic radiation compared to WT mice.^98^ The observed protection was highlighted by reduced radiation‐induced lung injury and production of pro‐inflammatory cytokines in cGAS and STING‐deficient mice.^98^ Similar upregulation and phosphorylation of STING following radiation was also observed in primary Kupffer cells.^95^ In a model of radiation‐induced liver damage, STING‐deficient mice showed reduced liver damage and reduced systemic levels of ALT and AST. Another study reported that treatment of mice with H151 (a STING antagonist) rescued liver damage, reduced levels of ALT and AST and, importantly, improved survival.^95^ Mechanistically, STING may promote radiation‐induced DNA damage, ROS production, and cell death.^105^ Interestingly, radiation‐induced STING activation and IRF3 signaling promotes activation of the NLRP3 inflammasome and pyroptosis, suggesting crosstalk of cGAS/STING signaling and the NLRP3 inflammasome activation.^95^, ^98^

The role of CLRs and RLRs in acute radiation syndrome remains unknown, and studies examining the role of these sensors in acute radiation syndrome are clearly warranted. In general, the role of innate immune sensors in radiation‐induced damage is an active area of research, and further investigation is needed to fully understand the mechanisms involved.

## 5 RADIATION AND MECHANISMS OF CELL DEATH

**FIGURE 4:**
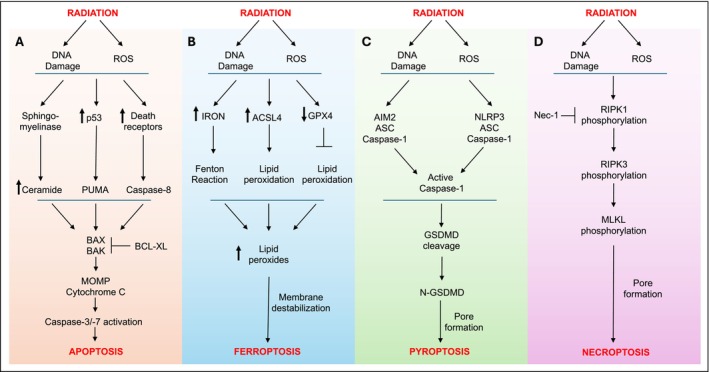
Different modalities of cell death induced by radiation. Radiation promotes cell death by promoting DNA damage and production of ROS via radiolysis of water. The specific cell death pathways that are induced by radiation include: (A) Apoptosis: Radiation induces production of ceramides, activation of p53, and death receptors, all of which can ultimately result in mitochondrial destabilization, caspase‐3/−7 activation, and induction of apoptotic cell death. (B) Ferroptosis: Radiation promotes accumulation of iron, increase in ACSL4 and decrease in GPX4, all of which ultimately leads to increased lipid peroxidation, membrane destabilization, and ferroptosis. (C) Pyroptosis: Radiation‐induced DNA damage and ROS production promote activation of AIM2 and NLRP3 inflammasome activation, which ultimately results in caspase‐1 activation. Active caspase‐1 promotes GSDMD cleavage leading to pyroptotic cell death. (D) Necroptosis: Radiation promotes RIPK1 and RIPK3 activation and phosphorylation, which ultimately promotes MLKL phosphorylation and necroptotic cell death.

Ionizing particles/energy from radiation sources, including x‐rays, γ‐rays, and ultraviolent rays, can promote single‐ and double‐strand DNA breaks, resulting in mutations and irreparable DNA damage. Additionally, this ionizing radiation promotes radiolysis of both intra and extracellular H_2_O, generating ROS. Together, the accumulating DNA damage and ROS promote cell death. Radiation‐induced cell death was originally primarily attributed to apoptosis; however, this was incorrect due to our lack of understanding of other forms of cell death. Recent advances in radiation research have uncovered several other modalities of non‐apoptotic inflammatory cell death, including necroptosis, pyroptosis, and ferroptosis. In this section, we will discuss the molecular mechanisms involved in regulating all modalities of radiation‐induced cell death (Figure 4).

### 5.1 Apoptosis

Apoptosis is a silent, non‐immunologic programmed cell death and the major cell death pathway for normal cellular turnover in the human body. First coined by Willie et al. in 1972, apoptosis is morphologically defined by its hallmark cell shrinkage, nuclear condensation, and membrane blebbing.^106^ Apoptosis can be induced by the cell extrinsic pathway via death receptors or the cell intrinsic pathway via mitochondrial destabilization.^107^, ^108^ The cell extrinsic pathway is activated when members of the tumor necrosis factor receptor (TNF‐R) family are activated, resulting in subsequent activation of caspase‐8.^108^ In contrast, cell intrinsic signaling is initiated by signals that activate BAX/BAK which form pores in the mitochondria to release cytochrome C and activation of caspase‐9.^108^ Both cell extrinsic and intrinsic pathways culminate in common activation of executioner caspases, caspase‐3 and caspase‐7, that ultimately drive apoptosis.^108^ The role of apoptosis in radiation‐induced cell death has been extensively studied and reviewed in detail.^107^, ^109^, ^110^, ^111^, ^112^, ^113^, ^114^ Herein, we will highlight the importance of major components of the apoptotic pathway in regulating radiation‐induced cell death (Figure 4A).

One of the major pathways that promotes radiation‐induced apoptosis and has been studied in detail involves ceramide synthesis (Figure 4A). Sphingomyelinase mediates hydrolysis of the phosphodiester bond of membrane sphingomyelin to generate ceramides.^115^, ^116^ Radiation promotes the generation of ceramides within minutes of exposure. One study reported that addition of ceramide analogs alone induced apoptosis of endothelial cells, demonstrating ceramides as direct inducers of apoptosis.^116^ Importantly, radiation of membrane preparations devoid of nuclear material also promoted hydrolysis of sphingomyelin into ceramides, suggesting a DNA‐break independent mechanism of radiation‐induced apoptosis. Studies by Michael et al.^117^ and Chmura et al.^118^ showed that sensitivity of lymphoma cell lines to radiation directly correlates with ceramide accumulation following radiation.^117^, ^119^ BL30A and HL‐60 cells that produce several‐fold higher ceramide levels 10 min following radiation were sensitive to radiation.^119^ In contrast, radiation failed to induce ceramide production in BL30K, BL29, BL36, and M059K cell lines, which are resistant to radiation‐induced apoptosis.^119^ Similar resistance to radiation was observed in lymphoblasts from Neimann‐Pick patients who have an inherited deficiency of sphingomyelinase required for ceramide generation.^118^ In this same study, mice deficient in sphingomyelinase failed to generate ceramides following radiation in vivo, as demonstrated by ceramide levels in the lungs at various time points and doses of radiation. Apoptotic cell death in several tissues, including lungs, spleen, and thymus, were significantly reduced in sphingomyelinase KO mice compared to WT controls. More recently, blocking ceramides using anti‐ceramide antibodies has been demonstrated to both protect and mitigate radiation‐induced death in a model of gastrointestinal acute radiation syndrome.^120^, ^121^, ^122^ Administration of anti‐ceramide antibody 15 min prior (radioprotection) or 24 h after (radiomitigation) 15 Gy WBR provided greater than 60% survival compared to no survival in control groups.^121^, ^122^ These studies demonstrate an important role for DNA damage‐independent ceramide accumulation in promoting radiation‐induced apoptosis and highlight ceramide blockade as a potential therapeutic target to treating acute radiation syndrome.

Another major signaling node at the center of radiation‐induced apoptosis is p53 (Figure 4A).^123^, ^124^ Radiation‐induced DNA damage induces translation of p53, resulting in higher expression of p53 in radiation‐exposed MCF7 cells and tissues as early as 10 min post‐radiation.^125^ Similar upregulation of p53 was observed in radiation‐exposed thymocytes at 1 and 2 h.^126^ More importantly, p53 is specifically required for radiation‐induced apoptosis in vivo.^126^ Immature thymocytes isolated from mice lacking p53 were highly resistant to radiation‐induced apoptosis but showed similar apoptosis in response to dexamethasone or glucocorticoid,^126^, ^127^, ^128^ demonstrating a specific role for p53 in promoting apoptosis in response to radiation. Radiation‐induced malformations in embryos have been linked to defective apoptosis,^129^ and p53‐deficient mice have demonstrated increased resistance to 1, 3, and 4 Gy radiation‐induced malformations when compared to p53‐sufficient WT controls.^130^ Interestingly, the role of p53 in radiation may be different depending on the organs or tissues that are affected. P53‐deficient mice treated with doses below 10 Gy that are known to cause hematopoietic failure have shown increased protection to lethal WBR when compared to WT controls.^131^, ^132^ Similar results were also observed with chemical inhibition of p53 (by pifithrin‐α) during 6–9 Gy WBR of BALB/c and C57BL/6 mice, that is, pifithrin‐α treatment provided significant protection from morbidity and mortality associated with 8 Gy radiation.^132^, ^133^ Interestingly, the protective effect of p53‐deficiency was not only lost when radiation doses were higher than 10 Gy (known to cause gastrointestinal issues), but p53‐deficient mice did much worse than controls. At radiation doses exceeding 10 Gy, p53‐deficient mice were highly sensitive and died with more rapid kinetics compared to controls. Others have shown that mice with gut‐specific deletion of p53 (p53‐floxed × Villin‐Cre mice) were highly sensitive to subtotal body irradiation targeting the gut area.^134^, ^135^ The role of p53 in promoting radiation‐induced apoptosis is clear and evident from the existing body of literature. However, concluding a positive role for p53 inhibition must be taken with caution given its clear opposing roles in hematopoietic vs. gastrointestinal tissues.

p53 promotes activation of cell‐intrinsic cellular components such as PUMA, BAX, and BAK, which ultimately promote cytochrome C release from mitochondria and activation of caspases‐3/7 leading to cell‐intrinsic apoptosis (Figure 4A).^136^, ^137^ Like p53‐deficient cells, PUMA‐deficient thymocytes are protected from radiation‐induced apoptosis in vitro and in vivo.^138^, ^139^ Radiation‐induced deletion of lymphocytes observed in WT mice in vivo was shown to be rescued by PUMA‐deficiency, demonstrating a central role for PUMA in radiation‐induced apoptosis.^140^ PUMA deficiency also protected intestinal epithelial cells from radiation‐induced apoptosis.^141^, ^142^ More importantly, PUMA‐deficient mice have been shown to be significantly more resistant to 10 Gy WBR than control mice.^143^ In addition, and in contrast to p53, PUMA‐deficient mice were reported to be protected from gastrointestinal syndrome and showed significant delay in death following 15 and 18 Gy WBR.^141^, ^144^ These studies highlight PUMA as a potentially more effective target for ameliorating radiation‐induced apoptosis and toxicity, since both hematopoietic and gastrointestinal syndromes are mitigated in the absence of PUMA.

Downstream of PUMA, proteins such as BAX and BAK play important roles in promoting mitochondrial outer membrane permeabilization and release of cytochrome C. Radiation exposure promotes mitochondrial BAX and BAK accumulation, cytochrome C release, and caspase‐3 activation, all of which have been shown to be abrogated in PUMA‐deficient cells, underscoring the role of PUMA as a direct regulator of these downstream apoptotic molecules.^141^ In support of BAX and BAK as direct modulators of apoptosis, mice with conditional deletion of both BAX and BAK in the hematopoietic compartment were significantly more resistant than WT controls when exposed to lethal 12.5 Gy WBR.^134^ However, subtotal body irradiation (to examine gastrointestinal syndrome) of mice with double deletion of BAX and BAK in the gut epithelium led to death with similar kinetics as littermate controls. Interestingly, another study examining mice singly deficient in either BAX and BAK showed that mice lacking either BAX or BAK were highly resistant to radiation‐induced gastrointestinal syndrome and mortality.^145^ These studies provide convincing evidence that BAX and BAK play critical roles downstream of PUMA as promoters of radiation‐induced apoptosis and death.

Cytochrome C‐deficient mice are embryonic lethal; however, Li et al. reported that fibroblasts generated from the fetal livers of cytochrome C‐deficient embryos were normal and showed significant resistant to radiation‐induced cell death and activation of caspase‐3.^146^ In another report, mouse embryonic cells treated with chemical inhibitors of cytochrome C mitigated radiation‐induced caspase‐3/7 activation and cell death.^147^ More importantly, chemical inhibition of cytochrome C in mice provided at 10 min, 1 h, or 5 h following 9.25 Gy WBR provided significant survival advantage compared to controls, indicating that cytochrome C is a potential target as a radiomitigator in the treatment of acute radiation syndrome.

Cell extrinsic apoptosis occurs via engagement of death receptors Fas (CD95), TNF‐R, and TRAIL‐R. Sheard and colleagues reported that radiation promoted upregulation of Fas in several cancer cell lines in a p53‐dependent manner.^148^ In another study, radiation also promoted upregulation of FasL and activation of caspase‐8, caspase‐3, and apoptosis in medulloblastoma and glioblastoma cell lines D283, Daoy, and A172.^149^ Radiation‐induced cell death was reversed by blocking FasL with a blocking antibody or the pan‐caspase inhibitor zVAD‐fmk.^149^ In support of a role for caspase‐8, another study demonstrated that following radiation, caspase‐8 was activated in a p53‐dependent manner.^150^ Afshar et al. demonstrated a critical role for radiation‐induced caspase‐8 activation in promoting cell death; however, this cell death was p53‐independent.^151^ These studies demonstrate a role for cell extrinsic apoptosis in radiation‐induced cell death.

Executioner caspases, caspase‐3, and caspase‐7 are downstream of both cell extrinsic and cell intrinsic apoptosis induced by radiation. The roles of executioner caspases in radiation‐induced apoptosis have been demonstrated by the ability of the pan‐caspase inhibitor zVAD‐fmk to rescue from cell death.^152^ In particular, caspase‐3/7 is activated by radiation and can execute cell apoptosis. Radiation‐induced cell death of mouse embryonic fibroblasts (MEF) has been reported to be significantly reduced in caspase‐3/7 DKO cells.^153^ Another study of caspase‐3 or caspase‐7 single KO MEF showed that caspase‐3 plays a more dominant role in mitigating radiation‐induced apoptosis.^153^

### 5.2 Ferroptosis

Iron‐dependent cell death was observed in RAS‐mutant cancer cell lines sensitive to small molecule compounds such as erastin and RSL‐3, which were dubbed RAS‐selective lethal (RSL) for their ability to kill.^154^, ^155^, ^156^ In 2012, Dixon et al. demonstrated that this cell death was distinct from traditional cell death pathways (apoptosis, necroptosis, and pyroptosis) and coined the term “ferroptosis” to describe this unique cell death.^157^ Subsequent studies have described many specific features of the ferroptotic pathway, including the requirement for cysteine/glutamate antiporter system (xCT),^157^ glutathione peroxidase 4 (GPX4),^158^ acyl‐coA synthetase long chain family member 4 (ACSL4),^159^, ^160^ ROS,^157^ lipid peroxidation,^161^, ^162^, ^163^ and ultimately localization of ninjurin1 (NINJ1) to the cell membrane.^164^

One of the major hallmarks of ferroptosis includes lipid peroxidation that eventually compromises the cell membrane integrity.^157^ Radiation‐induced lipid peroxidation has been observed for decades, well before ferroptotic cell death was identified.^165^ However, mechanisms of radiation‐induced lipid peroxidation that eventually lead to ferroptosis are only beginning to be understood (Figure 4B).

Accumulation of systemic and intracellular iron can promote chemical reactions that eventually lead to lipid‐ROS accumulation and ferroptosis.^157^, ^166^ Radiation exposure increases intracellular iron in cells in both in vitro and in vivo systems. Analysis of serum samples from several astronauts living in the International Space Station from 50 to 247 days, thus exposed to higher levels of ionizing radiation, demonstrated an increase in serum iron levels.^167^ More directly, in a controlled study, radiation treatments were shown to increase systemic levels of iron in the serum of breast cancer patients.^168^ Furthermore, radiation has been shown. to increase free intracellular iron levels in several in vitro and in vivo model systems.^169^, ^170^, ^171^, ^172^ Mechanistically, radiation can promote free iron release by (1) affecting the iron storage capacity of ferritin by reducing oxidized iron,^173^ (2) promoting proteolysis of ferritin to release free iron within the intracellular milieu,^170^, ^171^ and (3) radiation‐induced hemorrhage in bone marrow.^174^

Radiation upregulates ACSL4, represses GPX4 expression, and promotes ferroptosis in several cancer cell lines.^175^, ^176^ Additionally, exposure of mice to radiation induced greater expression of several ferroptosis promoter genes and repression of several ferroptosis inhibitor genes in several organs in vivo.^177^ Specifically, greater upregulation of ACSL4 and repression of GPX4 was observed in the small intestine. Along with increased expression of ACSL4 and repression of GPX4 following radiation, which is correlated with induction of ferroptosis, lipid peroxidation measured by examining MDA levels^178^ was also increased in tissues following radiation exposure in vivo.^177^

Polyunsaturated fatty acids (PUFAs) in phospholipids are prone to oxidation, and membrane lipid concentration of PUFA can directly correlate with levels of lipid peroxidation observed during radiation.^165^ One of the major enzymes that regulates PUFA levels in cells is ACSL4, which is directly involved in increasing and enriching PUFAs in the cell membrane.^159^, ^179^ Several studies have shown that ACSL4 levels are increased in cells following radiation exposure.^175^, ^177^ Thus, radiation increases ACSL4 levels, which catalyzes lipid peroxidation to promote ferroptosis. Studies have reported that short interfering RNA‐mediated deletion or chemical inhibition of ACSL4 in cancer and intestinal epithelial cell lines protects cells from radiation‐induced ferroptosis in vitro.^176^, ^180^ Finally, treatment of mice with troglitazone (ACSL4 inhibitor)^159^ rescued radiation‐induced lipid peroxidation and tissue damage.^177^ Mechanistically, radiation has been shown to cause activation of STAT1/IRF1 signaling axis in intestinal epithelial cells, which in turn promoted ACSL4 upregulation to regulate ferroptosis.^180^

GPX4‐deficient mice are embryonic lethal at around E7.5, indicating the importance of this protein in maintaining cellular homeostasis.^181^ Studies using conditional deletion of GPX4 have demonstrated the importance of GPX4 in maintaining tissue homeostasis by preventing cell death.^182^, ^183^ While it is not possible to study *Gpx4* ^−/−^ mice due to embryonic lethality, *Gpx4* ^+/−^ mice are born healthy and indistinguishable from *Gpx4* ^+/+^ littermate controls.^181^ Importantly, GPX4 protein and mRNA levels in several tissues were reported to be reduced by approximately 50% in *Gpx4* ^+/−^ compared to *Gpx4* ^+/+^ mice.^181^ *Gpx4* ^+/−^ mice showed increased sensitivity and susceptibility to 10 Gy radiation exposure, demonstrating a radioprotective role for GPX4. Radiation has been shown to reduce GPX4 expression in various in vitro and in vivo model systems.^171^, ^172^, ^176^, ^177^, ^184^, ^185^ Mechanistically, radiation could affect several factors that are known to limit both GPX4 expression and function. Indeed, radiation has been shown to repress SLC7A11 expression, a component of the xCT transporter system that directly regulates GPX4 levels.^186^, ^187^ Radiation also reduces glutathione levels required to maintain GPX4 activity and function.^186^, ^188^, ^189^

Thus, the current literature is very supportive of ferroptosis as one of the major cell death pathways induced by radiation. Studies targeting molecules of the ferroptosis pathway in combination with other major cell death pathways may lead to superior protection against radiation‐induced morbidity and mortality.

### 5.3 Necroptosis

Stimulation of death receptors (TNF‐R family members) activate extrinsic apoptosis that requires caspases. While inhibition of caspases rescues death receptor‐induced apoptosis (example, TNF + zVAD), cells still die, albeit by necrotic cell death.^190^ In 2005, Yuan and colleagues demonstrated that this necrotic cell death could be specifically inhibited by a small molecule inhibitor, necrostatin‐1 (Nec‐1), a specific inhibitor of receptor‐interacting protein kinase (RIPK) 1, and they termed this novel form of cell death necroptosis.^191^ Since then, we have seen a tremendous amount of research devoted to understanding the molecular mechanisms that drive necroptosis.^192^ In particular, the central players that drive necroptosis include RIPK1,^193^ RIPK3,^194^ and the ultimate executioner of necroptotic cell death, mixed lineage kinase domain‐like protein (MLKL).^195^

One of the first studies to investigate the contribution of necroptosis to radiation‐induced cell death examined the ability of RIPK1 inhibitor Nec‐1 to rescue radiation‐induced cell death in several thyroid and adrenocortical carcinoma cell lines (Figure 4C).^196^ Radiation‐induced cell death in TPC‐1, 8505‐C, and SW13 cell lines was rescued by Nec‐1 treatment, suggesting a role for necroptosis.^196^ Interestingly, Nec‐1 did not rescue radiation‐induced cell death in H295R cells; further analysis of RIPK1 expression in the cancer cell lines showed that TPC‐1, 8505‐C, and SW13 cells expressed RIPK1, whereas H295R cells did not. Importantly, Nec1 treatment also rescued proliferation in human papillary thyroid carcinoma tissue samples, demonstrating the relevance of necroptosis in tumor cells ex vivo. Similarly, radiation‐induced phosphorylation of RIPK1, RIPK3, and MLKL in HIEC, MCF‐7, and MDA‐MB‐2331 cancer cells, and radiation‐induced death of these cancer cells were reversed by addition of Nec‐1or siRNA‐mediated knockdown of MLKL.^197^, ^198^ In support of the role for necroptosis in radiation‐induced cell death, another independent study demonstrated radiation‐induced RIPK3 phosphorylation in the small intestine of mice exposed to 9.5 Gy radiation, which was reversed by Nec‐1 treatment.^199^ More importantly, Nec‐1 treated mice demonstrated significant survival following 9.5 Gy lethal whole‐body radiation when compared to vehicle treated controls.

In contrast, necroptosis was reported not to be involved in ultraviolent B radiation‐induced death of keratinocytes as demonstrated by lack of phosphorylated RIPK3 and MLKL.^200^ More importantly, another study reported that overall survival of RIPK3‐deficient mice following 6.7–7.8 Gy WBR was similar to littermate controls.^201^ In addition, both RIPK3‐deficient and littermate controls succumbed to 15.9 Gy sublethal body irradiation at a similar rate. These studies argue that necroptosis may not be involved in radiation‐induced toxicity in mice and careful examinations of variations of different model systems are required to rule in or rule out the contribution of necroptosis in radiation‐induced morbidity and mortality.

### 5.4 Pyroptosis

The term inflammasome, a multimeric protein complex consisting of a cytoplasmic sensor, a bipartite adaptor molecule ASC (apoptosis‐associated speck‐like protein containing a CARD), and cysteine protease caspase‐1, was first coined by Tschopp's research group.^202^ The inflammasome complex promotes activation of caspase‐1 which causes: (1) maturation of pro‐inflammatory cytokines IL‐1β and IL‐18; and (2) cleavage of GSDMD to release N‐terminal GSDMD that initiates inflammatory cell death.^203^ Cookson and Brennan named this specific form of caspase‐1‐dependent inflammatory cell death as pyroptosis.^204^, ^205^ The contribution of pyroptosis to radiation‐induced cell death has not been investigated until recently. Several cytoplasmic sensors have been demonstrated to be involved in radiation‐induced inflammasome activation and pyroptosis (Figure 4D).

One of the first studies that demonstrated involvement of inflammasome activation following radiation exposure was published in 2015 by Stoecklein et al.^100^ Using a flow cytometric approach to detect active caspase‐1, the authors demonstrated that radiation promoted caspase‐1 activation in a dose‐dependent manner in almost all immune cells examined in the spleen at day 1 post‐radiation.^100^ Importantly, the kinetics of caspase‐1 activation correlated with observed cell death, and this radiation‐induced cell death was attenuated in caspase‐1‐deficient mice.

Bo et al. showed that radiation‐induced DNA damage is sensed by DNA sensor AIM2, which formed AIM2 inflammasome and activated caspase‐1 and pyroptotic cell death in vitro and in vivo.^88^ As such, mice deficient in AIM2, ASC, and caspase‐1 are highly resistant to lethal radiation. Furthermore, mice lacking caspase‐1 specifically in the gut epithelium are also protected from lethal radiation and gut epithelium cell death, when compared to WT controls, demonstrating a role for caspase‐1 in gut epithelial cells. This study highlighted a critical role for AIM2 inflammasome activation and pyroptosis in radiation‐induced cell death and toxicity. However, subsequent studies examining these key molecules in pyroptosis did not report similar roles. For instance, studies from Brickey and Daniel et al. examining the role of mice deficient in ASC, caspase‐1, and caspase‐11 failed to observe any pathogenic roles for these pyroptosis‐inducing molecules during lethal radiation.^91^, ^206^

Several studies have unequivocally demonstrated that radiation exposure promotes pyroptotic cell death both in vitro and in vivo. So far, there is evidence for both NLRP3 and AIM2 sensors as potential regulators of radiation‐induced pyroptosis. However, the requirement of pyroptosis for radiation‐induced morbidity and mortality has been contested. Thus, well‐controlled studies are warranted to sort out some of these discrepancies regarding the role of pyroptosis in radiation‐induced toxicity.

## 6 CONCLUSIONS

Radiation is all around us, and we are constantly exposed to radiation that includes radiation from earth (e.g., radon gas) and space (e.g., cosmic rays). In addition, we are exposed to radiation for medical purposes, including X‐rays and CT scans for diagnostic purposes, and radiation therapy to treat cancers. Furthermore, our reliance on nuclear energy for generating electricity will only increase in the future – which also increases our chances of accidental radiation exposure. Lastly, warfare activities pose a direct risk of radiation exposure for the citizens of our world. The past couple of decades have seen tremendous progress in research aimed at understanding how radiation kills cancer cells and, not surprisingly, radiation is commonly used as a therapeutic strategy to treat several cancers today. However, our understanding of radiation exposure on the immune system and the subsequent sequalae of radiation exposure remain poorly understood. Research to understand the direct effects of radiation exposure is much needed and will unravel several novel pathways that can be targeted to mitigate its harmful effects.

## AUTHOR CONTRIBUTIONS

S.S. wrote the first draft of the manuscript. P.G. wrote, edited, and finalized the manuscript. All authors have read and agreed to the published version of the manuscript.

## CONFLICT OF INTEREST STATEMENT

The authors declare no conflicts of interest.

## Data Availability

Data sharing not applicable – no new data generated.
